# IRX5 Promoted SREBP1‐Mediated de Novo Fatty Acid Synthesis via HMGN4 in Hepatocellular Carcinoma

**DOI:** 10.1111/jcmm.70441

**Published:** 2025-04-10

**Authors:** Liying Zhu, Yongjie Xu, Changyudong Huang, Chengcheng Li, Yiqiong Zhang, Xing Li, Wei Pan, Zhu Zeng

**Affiliations:** ^1^ Center for Clinical Laboratories the Affiliated Hospital of Guizhou Medical University Guiyang People's Republic of China; ^2^ School of Basic Medical Sciences/School of Biology & Engineering Guiyang Guizhou People's Republic of China; ^3^ Guizhou Prenatal Diagnosis Center the Affiliated Hospital of Guizhou Medical University Guiyang People's Republic of China; ^4^ School of Public Health, the key Laboratory of Environmental Pollution Monitoring and Disease Control Ministry of Education, Guizhou Medical University Guiyang China; ^5^ Guizhou University of Traditional Chinese Medicine Guiyang Guizhou People's Republic of China

**Keywords:** de novo lipogenesis, hepatocellular carcinoma, IRX5, nuclear translocation

## Abstract

Hepatocellular carcinoma (HCC), a prevalent malignant tumour, ranks highly in both morbidity and mortality, and its prevention and treatment need further studies. The transcription factor iroquois homeobox 5 (IRX5) plays an essential role in HCC, whereas little is known about its exact functions and underlying mechanisms in tumour metabolism reprogramming. Besides, as a transcription factor that mainly locates in nuclei, IRX5 lacks a nuclear localisation sequence, which makes uncovering the mechanism of IRX5 translocating into the nuclei of great significance. Here, we first found that both IRX5 and HCC development are highly expressed; IRX5 accelerates de novo fatty acid synthesis and promotes cancer cell proliferation and progression. Moreover, the GST pull‐down combined with GC/MS experiments identified an interaction between IRX5 and high‐mobility group nucleosomal binding domain 4 (HMGN4). Immunofluorescence analysis showed that IRX5 and HMGN4 colocalised within the nucleus. Coimmunoprecipitation further confirmed their direct interaction. The elevated expression of HMGN4 enhanced the nuclear transport of IRX5. Taken together, our observations suggest that HMGN4 driving IRX5 nuclear translocation promotes HCC development via de novo fatty acid synthesis reprogramming.

## 1 Introduction

Hepatocellular carcinoma (HCC) is a common primary human liver malignancy worldwide, and its mortality rate ranks fourth among malignant tumours [1]. In patients with liver diseases of other aetiologies, metabolic syndrome and type 2 diabetes are associated with an increased risk of HCC [2]. These findings indicate that altered lipid metabolism plays critical roles in HCC. However, the molecular mechanism has not yet been fully elucidated.

The accumulation of lipids is crucial for the survival and metastasis of tumour cells [3]. The metabolism of lipids, specifically their synthesis, is significantly altered and upregulated in cancer, and the uptake and storage of exogenous lipids are also increased [4]. The production of cholesterol and complex lipids, such as phospholipids and triglycerides, depends on fatty acid (FA) synthesis. In addition to functioning as signalling molecules, storage compounds, and energy sources, FAs are structural components of the cell membrane, and they are all vital for cancer cell growth. In contrast to normal cells, cancer cells tend to synthesise FA de novo instead of relying on exogenous sources [5]. In a study that analysed global gene expression profiles of HCC, researchers found that FA synthesis‐related genes are universally upregulated in HCC tissues compared to noncancerous liver tissues [6]. De novo FA synthesis involves key enzymes [5, 7, 8], such as fatty acid synthase (FASN), acetyl‐CoA carboxylase 1 (ACC1), ATP citrate lyase (ACL), and stearoyl‐coenzyme A desaturase 1 (SCD1), which are highly expressed in HCC. The transcription factor sterol regulatory element‐binding protein 1 (SREBP1) regulates FA synthesis [9]. Therefore, finding new regulators of SREBP1 will provide potential therapeutic targets for HCC.

Iroquois Homeobox Gene 5 (IRX5) is a member of the Iroquois homeodomain protein family. We have previously shown that IRX5 regulates cell proliferation, migration, and invasion in HCC [10, 11]. According to accumulating research, IRX5 is also involved in the metabolism of cellular energy [12, 13, 14]. However, whether IRX5 is involved in de novo FA synthesis in HCC remains largely unknown. Here, we identified the regulatory role of IRX5 in FA synthesis.

## 2 Materials and Methods

All the methods are in accordance with relevant institutional guidelines and regulations, and all experiments were performed in accordance with the ARRIVE guidelines.

### 2.1 Cell Lines and Tissue Samples

The HepG2, SMMC7721 hepatocellular carcinoma, and HEK‐293T cell lines were obtained from the Chinese Academy of Sciences Cell Bank. Additionally, serum samples from 50 healthy individuals and 100 hepatocellular carcinoma patients, as well as four pairs of hepatocellular carcinoma tissues, were collected from the Affiliated Hospital of Guizhou Medical University between May 2019 and March 2020. A portion of all the tissues was fixed with 4% paraformaldehyde and embedded in paraffin, and the remaining tissues were stored at −80°C. All the patients provided signed written informed consent, and specimen collection was approved by the Medical Ethics Committee of the Affiliated Hospital of Guizhou Medical University (approval no. 2019‐170).

### 2.2 TCGA Profiles Analysis

We obtained RNA‐sequencing expression profiles (level 3) and corresponding clinical data for hepatocellular carcinoma from the TCGA dataset (https://portal.gdc.com). Survival differences between groups were assessed using the log‐rank test. The predictive accuracy of hepatocellular carcinoma mRNA was compared using the timeROC (v 0.4) analysis. For the Kaplan–Meier curves, *p*‐values, and hazard ratio (HR) with a 95% confidence interval (CI), we utilised log‐rank tests and univariate Cox proportional hazards regression. We employed the R software GSVA package for analysis, utilising the parameter method = ‘ssgsea’. The Spearman correlation was employed to analyse the correlation between genes and pathway scores of ‘Biosynthesis of unsaturated fatty acids’, ‘Fatty acid elongation’. All analyses were conducted using R (Foundation for Statistical Computing 2020), version 4.0.3. *p* value < 0.05 was considered statistically significant.

### 2.3 Animal Studies

The male BALB/c nude mice (4–6 weeks old) were purchased from the Laboratory Animal Services Center of Guizhou Medical University. Twenty mice were randomLy allocated into four groups: stable SMMC7721 cells (1 × 10^7^, 200 μL) transfected with pcDNA3.1, pcDNA3.1‐ IRX5, sh‐NC or sh‐IRX5 and were injected into the livers of the mice, and the mice were euthanised 8 weeks after injection. The animal experiments were approved by the Ethics Committee of Guizhou Medical University (Guizhou, China) (approval no. 1800360) and conducted in a manner that was consistent with scientific and ethical principles.

### 2.4 Plasmid Transfection

pcDNA3.1 and pcDNA3.1‐IRX5 were purchased from GenePharma (Shanghai, China). Flag‐HMGN4 expression plasmid was purchased from Guangzhou IGE Biotechnology Co. Ltd. (Guangzhou, China). The IRX5 knockdown plasmid was constructed by the research group. ViaFectTM Transfection Reagent was used for transfection according to the instructions.

### 2.5 GST Pull‐Down Combined With GC/MS Analysis

Glutathione Sepharose Beads Glutathione‐S‐transferase fusion proteins or GST alone were expressed in *Escherichia coli* and immobilised on glutathione Sepharose 4B beads (GE Healthcare). GST proteins were incubated with whole‐cell extracts of HepG2 cells. Unbound proteins were removed, and bound proteins were eluted and analysed by SDS‐PAGE, and gas chromatography tandem mass spectrometry (GC/MS) analysis was performed via Shanghai Yunke Bio‐Technology Co. Ltd (Shanghai, China).

### 2.6 Immunofluorescence (IF) Assay

Cells were seeded and transfected with NC or HMGN4 shRNA in 6‐well plates. After 48 h, the cells were collected and fixed with 4% paraformaldehyde, permeabilised with Triton X‐100, and blocked with blocking buffer (Beyotime). The samples were incubated with the primary antibodies anti‐HMGN4 and anti‐IRX5 (CST) and fluorescein‐labelled secondary antibody (CST), and then DAPI (Beyotime) was used to stain the nucleus. For semiquantitative analysis, ImageJ software was used to measure the fluorescence intensity.

### 2.7 Western Blotting

Total proteins were extracted by RIPA lysis buffer and PMSF (Solarbio, Beijing, China). The protein concentrations were determined by a BCA protein concentration determination kit (Solarbio, Beijing, China). The proteins were electrophoresed and transferred to polyvinylidene fluoride membranes (Merck Millipore, MA, USA). Then, the membranes were incubated with 5% skim milk for 2 h and incubated with antibodies against GAPDH (diluted 1:8000, Bioworld, China), β‐actin (diluted 1:2000, ProteinTech, Wuhan, China), IRX5 (diluted 1:500, Abcam, Cambridge, MA, USA), SREBP‐1 (diluted 1:3500, ProteinTech, Wuhan, China), ACC1 (diluted 1:750, ProteinTech, Wuhan, China) and FASN (diluted 1:800, ProteinTech, Wuhan, China). The membranes were stored at 4°C. The next day, the membranes were incubated in secondary antibodies (diluted 1:5000, Affinity Biosciences, Changzhou, China) at room temperature for 1.5 h. The luminescent liquid was dropped on the membranes and exposed with a gel imager (Peiqing Technology, Shanghai, China), and relative protein levels were quantified via ImageJ.

### 2.8 Luciferase Assay

The pGL3‐Basic Luciferase Reporter vector, along with the pRL‐TK and a 2.1‐kb SREBP‐1 promoter inserted into the pGL3‐Basic vector (pGL3‐Basic‐Homo‐SREBP1‐promoter‐2100), was obtained from Guangzhou IGE Biotechnology Co. Ltd. The activity of the SREBP‐1 promoter was adjusted by cotransfecting with the pRL‐TK plasmid. After 48 h posttransfection in the HEK‐293T cell line, the activities of firefly and Renilla luciferases were assessed via the Dual Luciferase Reporter Gene Assay Kit (Abbkine Scientific Co. Ltd).

### 2.9 Coimmunoprecipitation (Co‐IP)

Cell lysates were prepared using RIPA (Beyotime) or Pierce IP lysis buffer (Thermo Fisher Scientific) containing a protease inhibitor cocktail. Protein concentration quantification was performed by BCA assay (Beyotime). The membranes were blocked in 5% skin milk for 1 h and then incubated with primary antibodies overnight at 4°C. Horseradish peroxidase‐conjugated secondary antibody (Beyotime) was then added and incubated for 1 h at room temperature. Briefly, for Co‐IP, protein supernatants were incubated with Protein A/G PLUS‐Agarose (Santa Cruz Biotechnology) beads for 3 h at 4°C. The supernatants were transferred to new tubes, anti‐IRX5 or anti‐IgG antibodies were added, and the tubes were incubated for 1 h at 4°C. The mixtures were incubated with beads under rotary agitation overnight at 4°C and then centrifuged, and the supernatants were discarded. The beads were collected and washed three times. Then, 2× loading buffer was added to the beads, and the mixtures were incubated at 100°C for 10 min. The protein‐antibody complexes were analysed by immunoblotting.

### 2.10 Cell Oil Red O Staining

HepG2 cells were fixed with 4% paraformaldehyde for 15 min. An Oil Red O working solution was prepared in advance according to a stock solution:diluent ratio of 5:2, and this solution was incubated at room temperature for 10 min and filtered with a 0.45 μm filter. The filtered working solution was added to HepG2 cells and incubated for 30 min, and then the counterstain solution was added and incubated for 3 min. Water‐based mounting tablets were dropped onto the slide, and pictures were taken with an upright microscope.

### 2.11 Cell Triglyceride Assay

The total intracellular triglyceride levels were measured via triglyceride assay kit (Nanjing Jiancheng Bioengineering Institute). A standard curve was generated, and samples were added according to the instructions. The cellular triglyceride concentration was calculated according to the standard curve, and the cellular triglyceride content was normalised according to the protein amount (mg) in the sample.

### 2.12 Immunohistochemical Staining

The tissue sections were placed in a 55°C oven and incubated for 4 h, incubated in xylene solution for 10 min, incubated in new xylene solution for 10 min, and then incubated in absolute ethanol, 95%, 85% and 70% ethanol solutions for 5 min each. A 0.01 M sodium citrate solution was added to the sections, heated to boiling in a microwave oven, and thoroughly cooled at room temperature. After removing the water, a 3% H_2_O_2_ solution was added, and the sections were incubated in a humid box for 15–20 min. Then, 10% goat serum was gently dripped onto the sections, and the sections were incubated in a humid box at room temperature for 30 min. The tissue sections were incubated with antibodies against IRX5 (diluted 1:150, Atlas, USA), Ki‐67 (diluted 1:500, Abcam, Cambridge, UK), ACC1 (diluted 1:200, ProteinTech, Wuhan, China) and FASN (diluted 1:300, ProteinTech, Wuhan, China) and then stored at 4°C. The next day, the sections were incubated with an HRP‐conjugated goat anti‐rabbit secondary antibody for 30 min (diluted 1:1000, Bioword, USA), and then DAB was added for the colour reaction. The sections were incubated with 70%, 85% 95%, and absolute ethanol for 5 min for dehydration, incubated in xylene solution for 10 min, and then incubated in new xylene solution for 10 min. An upright microscope was used to observe the sections and capture images. Histoscore (H‐score) was employed for the semiquantitative analysis of immunohistochemistry.

### 2.13 Lipidomics Analysis

HepG2‐sh‐IRX5 cells were cultured for 48 h. The cells were collected in 1 mL methanol and thoroughly mixed by vortexing for 15 s, and then, the protein precipitate was pelleted at 140,000 g. The supernatant (100 μL) was stored at −80°C until lipidomics and analysis were conducted by Beijing Bio‐Tech Pack Technology Company Ltd.

### 2.14 Statistical Analysis

SPSS 19.0 software and GraphPad software were used for the statistical analyses. Measurement data that conformed to a normal distribution are expressed as the mean ± standard deviation. Comparisons between two groups were performed by *t* test, and comparisons among four groups were performed by one‐way analysis of variance. *p* < 0.05 was considered statistically significant.

## 3 Results

### 3.1 IRX5 Highly Expressed in HCC Serum and Tissues and Associated With Poor Survival

**FIGURE 1:**
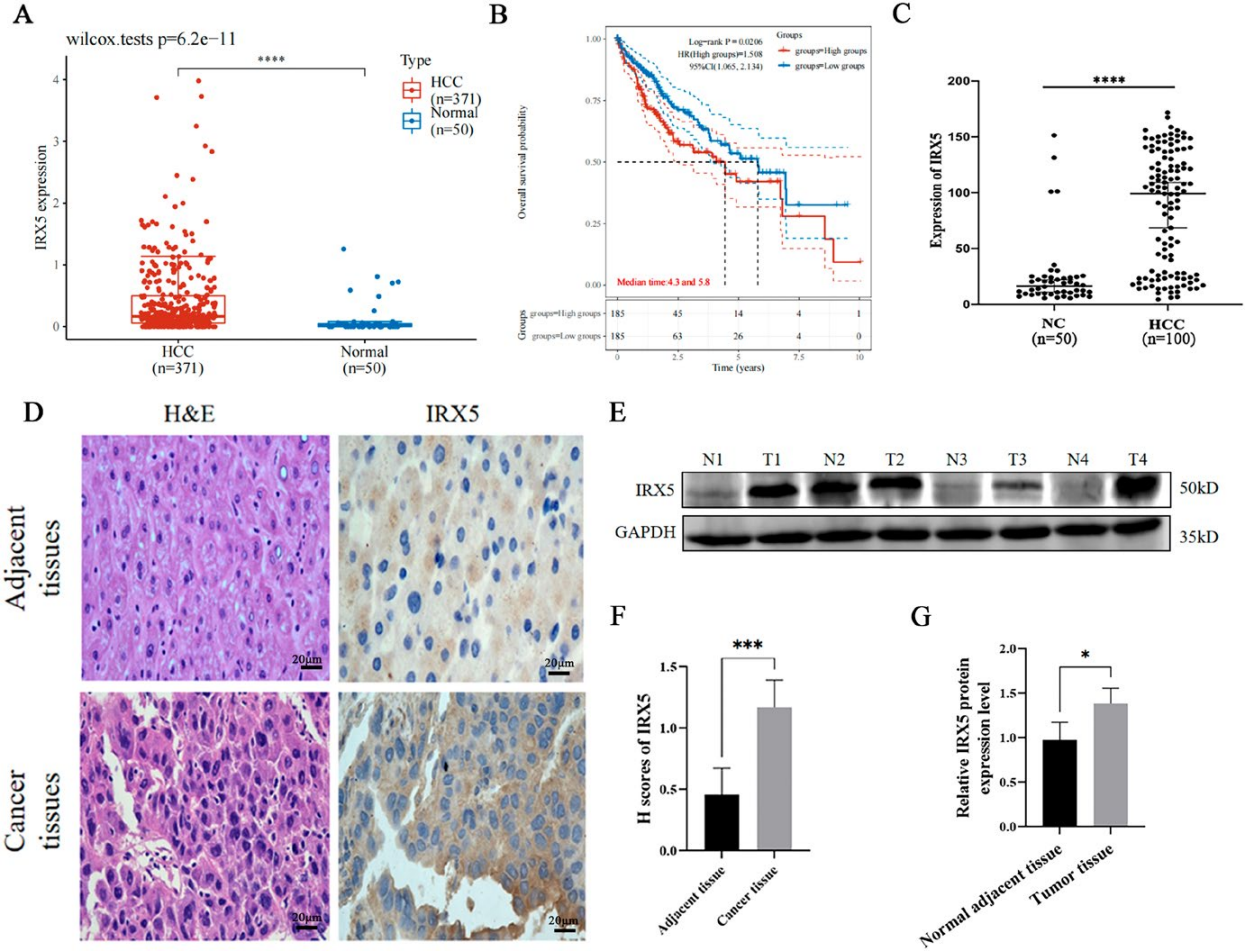
IRX5 is highly expressed in HCC serum and tissues and is associated with poor survival. (A) IRX5 mRNA expression level between HCC tissues (*n* = 371) and normal tissues (*n* = 50). (B) Kaplan–Meier survival analysis of the IRX5 signature from the TCGA dataset; comparison among different groups was made by log‐rank test. (C) The levels of IRX5 in 50 healthy females (NC) and 100 HCC serum samples were detected by ELISA analyses. (D) Representative images of H&E and immunohistochemical staining of IRX5. Western blot analysis. (E) Detection of IRX5 expression in four pairs of normal adjacent tissues (N) and tumour tissues (T) by Western blot. (F) H‐scores analysis of IRX5. (G) Western blot analysis. Comparison with normal adjacent tissues or NC; *indicates *p* < 0.05, ***indicates *p* < 0.001, while ****indicates *p* < 0.0001.

Through TCGA database analysis, we found that the expression of IRX5 mRNA in HCC tissue was significantly higher than that in Normal tissues (Figure 1A). Analysis of the Kaplan–Meier survival curve showed that high expression of IRX5 was a risk factor for poor prognosis of HCC (HR = 1.508), while the median survival time of patients with high expression of IRX5 was 4.3 years, and that of patients with low expression of HCC was 5.8 years (Figure 1B). Western Blot and IHC results showed that the expression of IRX5 was significantly increased in HCC cancer tissues (Figure 1C,D). The serum of 100 patients with liver cancer and 50 healthy controls was collected, and IRX5 was detected by ELISA. It was found that the concentration of IRX5 in the serum of patients with liver cancer was higher (Figure 1E). These results suggest that IRX5 is highly expressed in cancer tissues and that IRX5 may be involved in the occurrence and development of liver cancer. IRX5 is essentially a transcription factor that primarily functions within the cell nucleus to regulate gene expression and the development of multiple organs [15, 16]. It lacks a signal peptide, which makes it unable to be actively secreted extracellularly. However, during the progression of tumours such as liver cancer, tumour cells may undergo necrosis or apoptosis, leading to cell membrane rupture and the passive release of intracellular proteins like IRX5 into the bloodstream. The detection of IRX5 in serum may be related to this process of cell lysis. Additionally, inflammation in the tumour microenvironment and extracellular vesicles, such as exosomes, may also contribute to the transfer of IRX5 to the extracellular space, allowing its detection in the blood.

**FIGURE 2:**
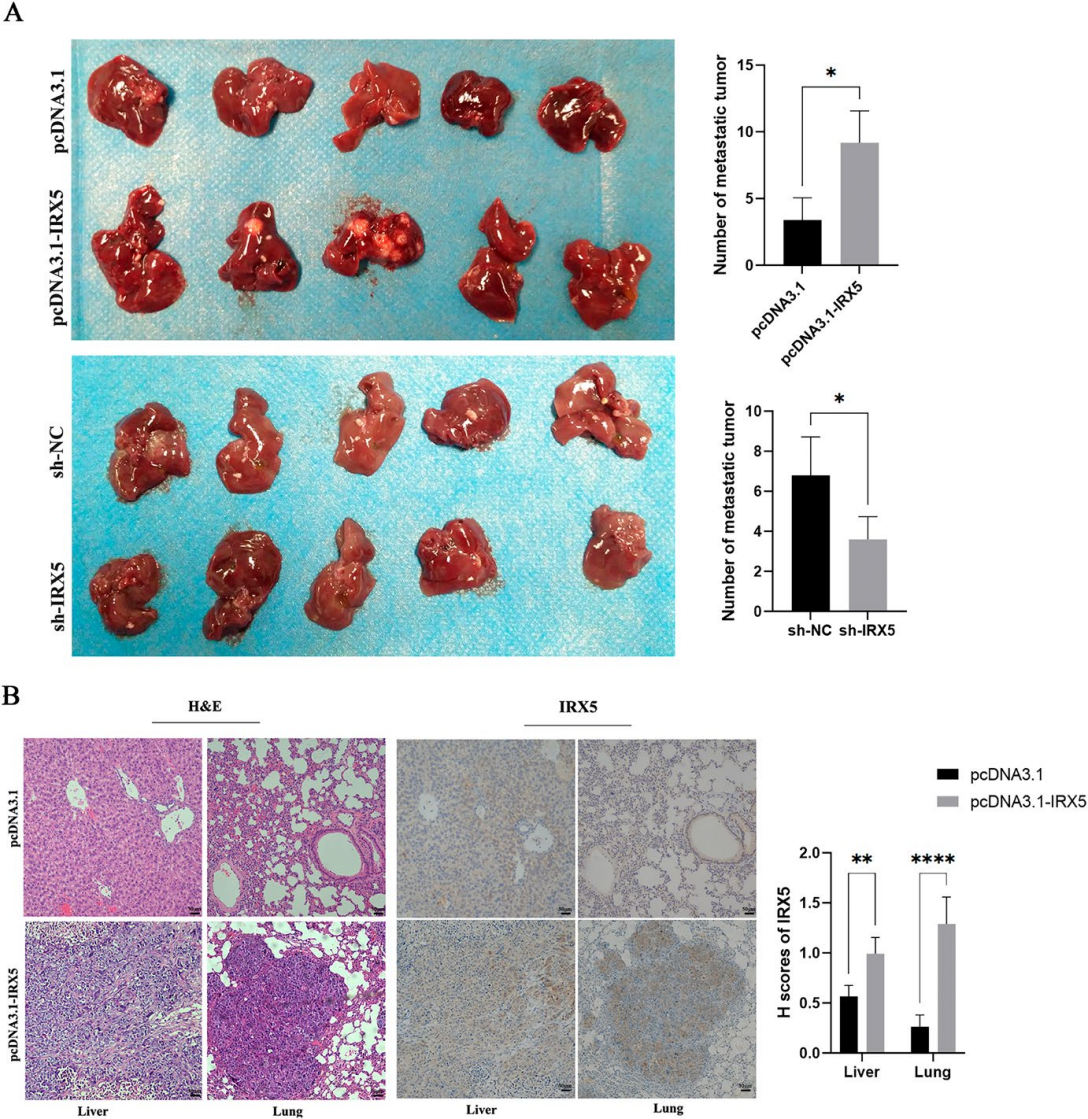
IRX5 promoted HCC tumour metastasis in vivo. (A) The livers collected from orthotopic tumour models were exhibited. Each group contained five nude mice; the number of metastatic foci was shown and quantified. (B) Representative images of H&E reveal tumour metastases in the liver and lung of nude mice, and immunohistochemical analyses were performed to detect the protein levels of IRX5; the H‐scores of IRX5 were shown and quantified. *indicates *p* < 0.05, **indicates *p* < 0.01, while ****indicates *p* < 0.0001.

To evaluate the impact of IRX5 on the metastasis of HCC in orthotopic tumour models, SMMC7721 cells transfected with pcDNA3.1, pcDNA3.1‐IRX5, sh‐NC or sh‐IRX5 were inoculated into the livers of male nude mice. Our results demonstrated that mice injected with pcDNA3.1‐IRX5 SMMC7721 cells exhibited a higher number of metastatic nodules in liver tissues compared to the vector control group, whereas the sh‐IRX5 group showed a reduction in metastatic nodules compared to its control group, sh‐NC (Figure 2A). Moreover, IRX5 overexpression enhanced the percentage of liver and lung metastasis compared with the control group by histopathological analysis with H&E staining, and the expression of IRX5 was also upregulated with immunohistochemical staining (Figure 2B).

### 3.2 IRX5 Regulated Signalling Pathway of Biosynthesis of Unsaturated Fatty Acids and Fatty Acid Elongation

**FIGURE 3:**
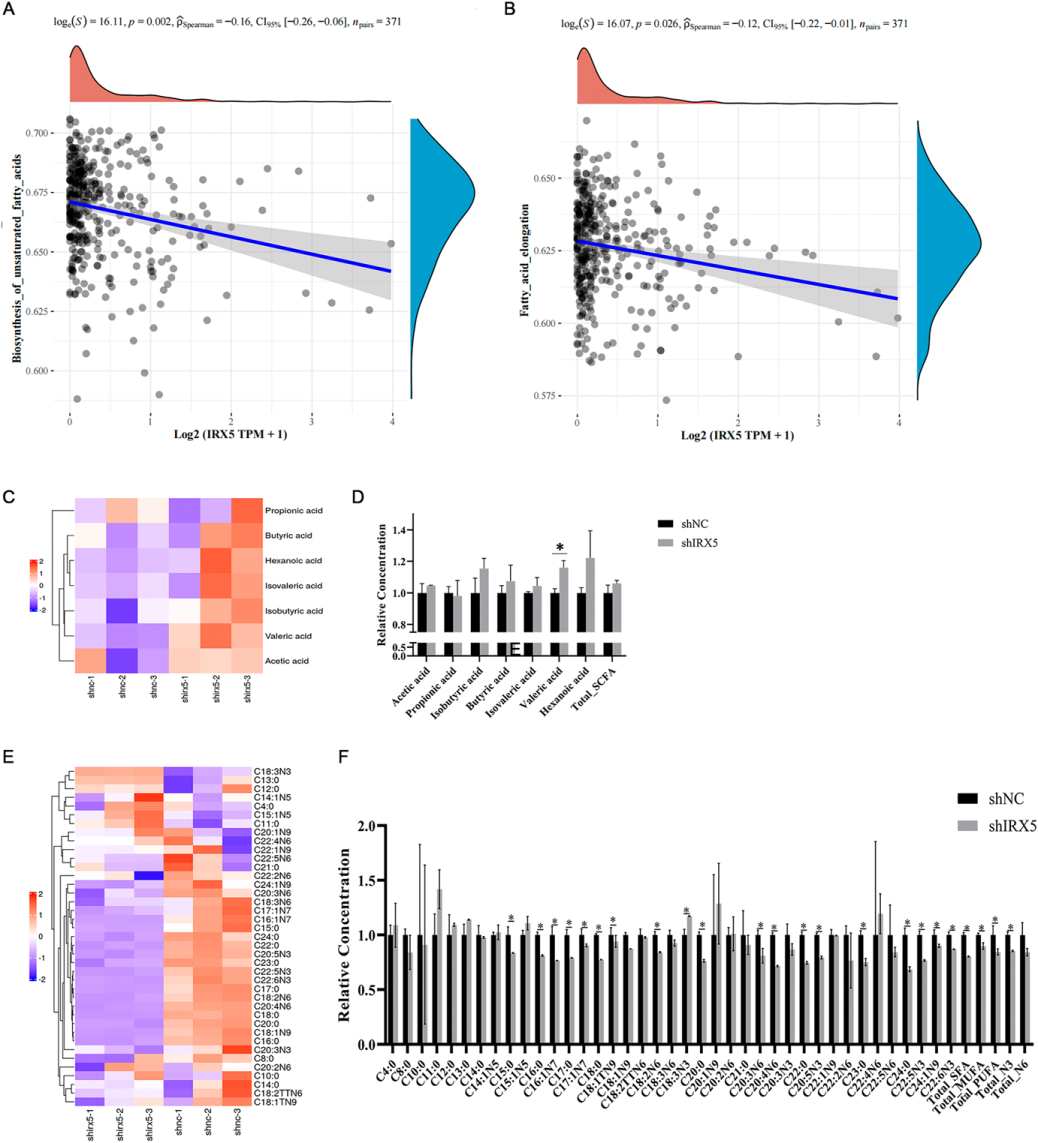
IRX5‐regulated signalling pathway of biosynthesis of unsaturated fatty acids and fatty acid elongation. (A) and (B) GSVA and Spearman correlation analyse of the relationship between IRX5 and biosynthesis of unsaturated fatty acids or fatty acid elongation signalling pathway. (C) and (D) Different fatty acid clustering heatmap. Red: fatty acids with high concentrations; blue: fatty acids with low concentrations. (E) and (F) Relative fatty acid concentrations. * indicates *p*  < 0.05.

Based on the analysis of the TCGA database, we found that the expression of IRX5 in hepatocellular carcinoma was related to the biosynthesis of unsaturated fatty acids and the fatty acid elongation signal pathway (Figure 3A,B). Additionally, we analysed fatty acid metabolism in Hepg2‐shIRX5 cells, and we found that the level of IRX5 was closely related to the concentrations of short‐chain and long‐chain fatty acids; among these fatty acids, the concentration of valeric acid (ValericAcid) was increased. The concentrations of total saturated fatty acids (TotalSFA) were decreased, and among unsaturated fatty acids, total monounsaturated fatty acids (TotalMUFA), total polyunsaturated fatty acids (TotalPUFA), fatty acids with the first unsaturated bond at the third position of the methyl end of the carbon chain (TotalN3), and fatty acids with the first unsaturated bond at the sixth position of the methyl end of the carbon chain were decreased (Figure 3C–F). These data prove that IRX5 may play an important role in regulating fatty acid metabolism in hepatocellular carcinoma cells and directly affect the expression of many fatty acid synthesis‐ and metabolism‐related genes.

### 3.3 IRX5 Promoted de Novo Fatty Acid Synthesis in HCC Cells

**FIGURE 4:**
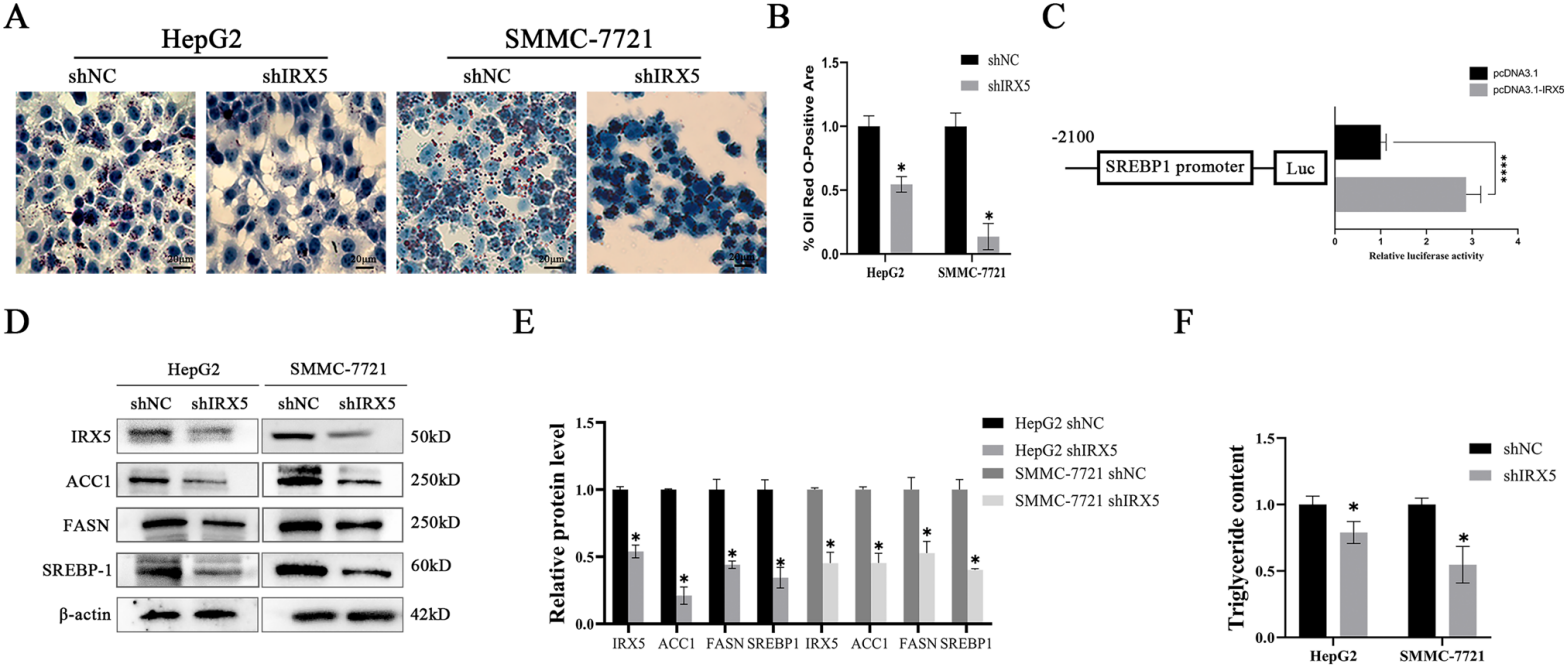
IRX5 promotes de novo fatty acid synthesis in HCC cells. (A) Representative Oil Red O staining images of IRX5‐knockdownHepG2 and SMMC7721 cell lines or normal control cell lines (200×). (B) % Oil Red O‐positive area, AA (%) = positive area/total cell area×100%; (C) The relative luciferase activities were measured in HEK‐293 T, (D) Representative Western blot. (E) Western blot analysis. (F) Intracellular triglyceride levels. *indicates *p* < 0.05, while ****indicates *p* < 0.0001.

IRX5 has been shown to promote de novo fatty acid synthesis in hepatocellular carcinoma (HCC) cells. Previous data suggested that IRX5 plays a role in disordered fatty acid metabolism, but the precise relationship was unclear. We focused on two hepatoma cell lines with high IRX5 expression for further investigation. Oil Red O staining indicated that knocking down IRX5 reduced FFA‐induced lipid droplet accumulation (Figure 4A,B). To explore whether IRX5 acts as a transcription factor regulating SREBP1‐dependent fatty acid metabolism, luciferase assays were conducted. The results revealed a significant increase in relative luciferase activity in HEK‐293T cells transfected with pcDNA3.1‐IRX5 compared to the control vector (Figure 4C). Since fatty acid synthesis is regulated by various rate‐limiting enzymes [5, 7, 8, 17, 18], we also examined the expression of key enzymes following IRX5 downregulation. Interestingly, the protein levels of ACC1, FASN, and SREBP1 were significantly decreased (Figure 4D,E), along with a reduction in intracellular triglyceride concentrations (Figure 4F). Collectively, these findings suggest that high IRX5 expression promotes de novo fatty acid synthesis in HCC cells.

**FIGURE 5:**
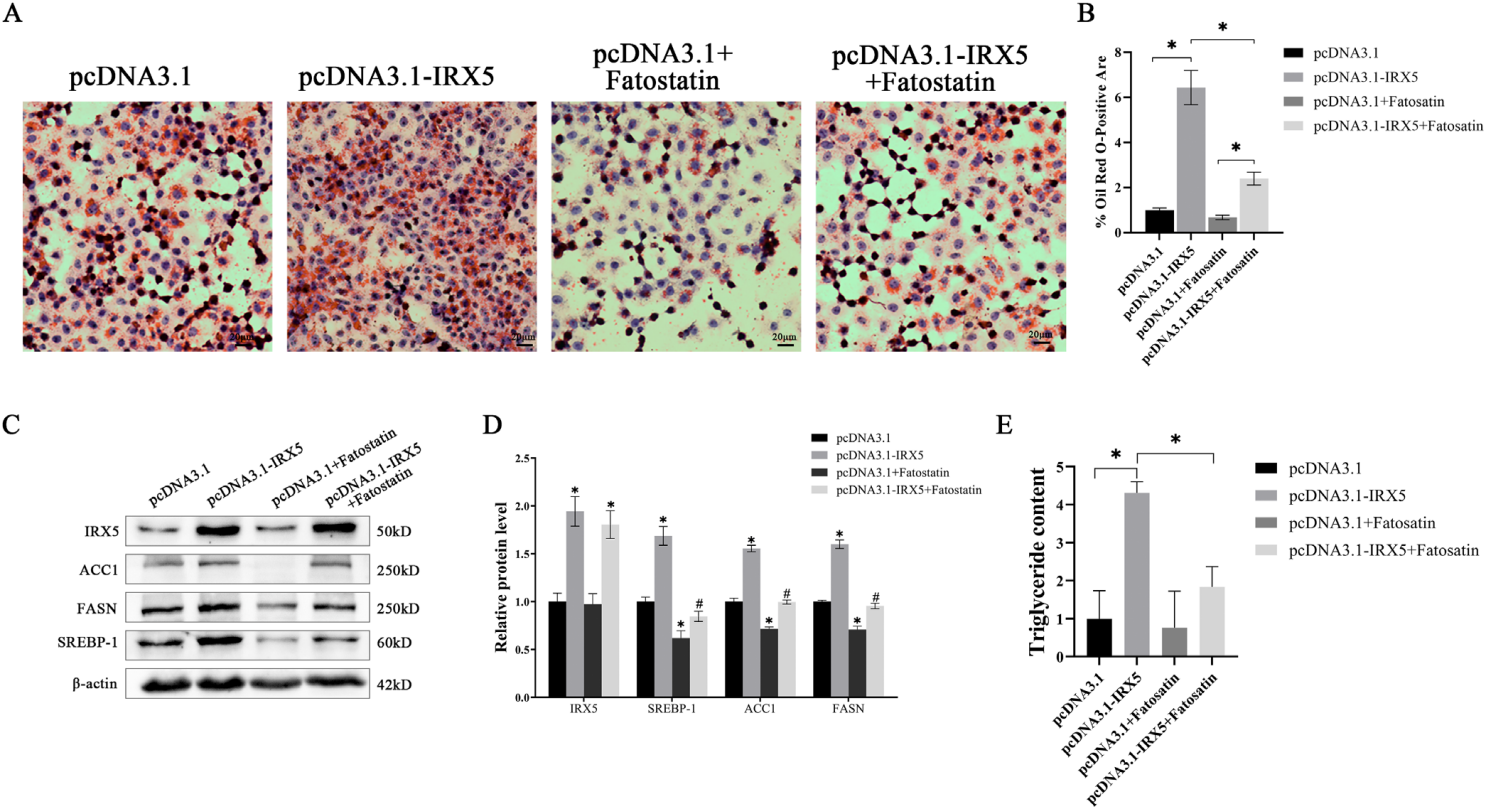
IRX5‐mediated promotion of de novo fatty acid synthesis was suppressed by fatostatin, an SREBP1 inhibitor. (A) Representative Oil Red O staining images of IRX5‐knockdown HepG2 and SMCC7721 cell lines or normal control cell lines (200×). (B) % Oil Red O‐positive area, AA (%) = positive area/total cell area×100%; (C) Representative Western blot; (D) Western blot analysis;(E) Intracellular triglyceride levels. *indicates *p* < 0.05.

Further, we demonstrated that IRX5 promotes SREBP1 expression to drive de novo fatty acid synthesis. Since SREBP1 is crucial for fatty acid biosynthesis, we investigated the relationship between IRX5 and SREBP1 by treating the cell model with the SREBP1 inhibitor fatostatin. Oil Red O staining revealed that fatostatin treatment diminished lipid droplet accumulation induced by high IRX5 expression (Figure 5A,B). Consistent with these results, fatostatin also reduced the expression of fatty acid synthesis‐related enzymes and intracellular triglyceride concentrations. Notably, however, treatment with the SREBP1 inhibitor did not alter IRX5 expression (Figure 5C–E). These data indicate that IRX5 acts as an upstream regulator of SREBP1, and while inhibiting SREBP1 reduces fatty acid synthesis, it does not affect IRX5 expression levels.

### 3.4 HMGN4 Directly Interacted With IRX5

**FIGURE 6:**
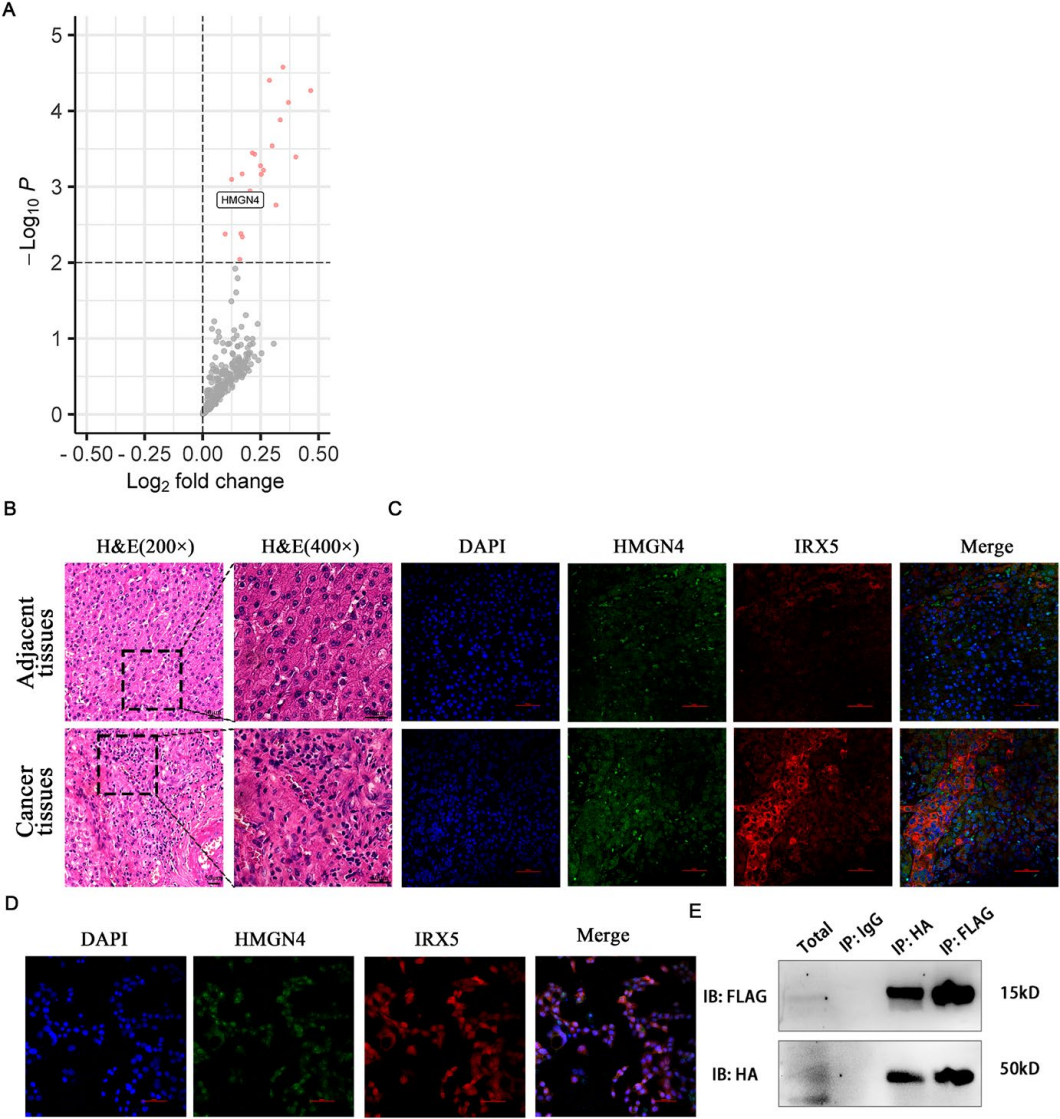
IRX5 interacts with HMGN4. After GST and GST‐IRX5 pull down, the associated proteins were analysed by LC–MS/MS. (A) Volcano plot showed proteins with significant differences between the GST and GST‐IRX5 groups. (B) Representative images of H&E of HCC tissue. (C) and (D) Representative immunofluorescence images of the colocalisation of IRX5 (red fluorescence)and HMGN4 (green fluorescence) in HCC tissues and HepG2 cell lines. (E) CoIP. HEK‐293T cells were transiently cotransfected with HA‐IRX5 and FLAG‐HMGN4 expression plasmids. Cytosolic extracts were incubated with the indicated Abs conjugated to protein A/G agarose beads, and then CoIP was performed.

To explore the proteins potentially involved in the nuclear transport of IRX5, we performed GST pull‐down assays combined with mass spectrometry, through which HMGN4 was identified as a candidate binding protein (Figure 6A). Subsequent analyses, including immunofluorescence colocalisation, were conducted in both cellular and animal models to verify the spatial association of IRX5 and HMGN4. The results consistently demonstrated colocalisation in both in vivo and in vitro models (Figure 6B–D). Additionally, coimmunoprecipitation assays further confirmed a direct binding interaction between IRX5 and HMGN4 in 293T cells (Figure 6E). These findings provide compelling evidence of both the colocalisation and binding relationship between IRX5 and HMGN4.

### 3.5 HMGN4 Regulated the Nuclear Translocation of IRX5

**FIGURE 7:**
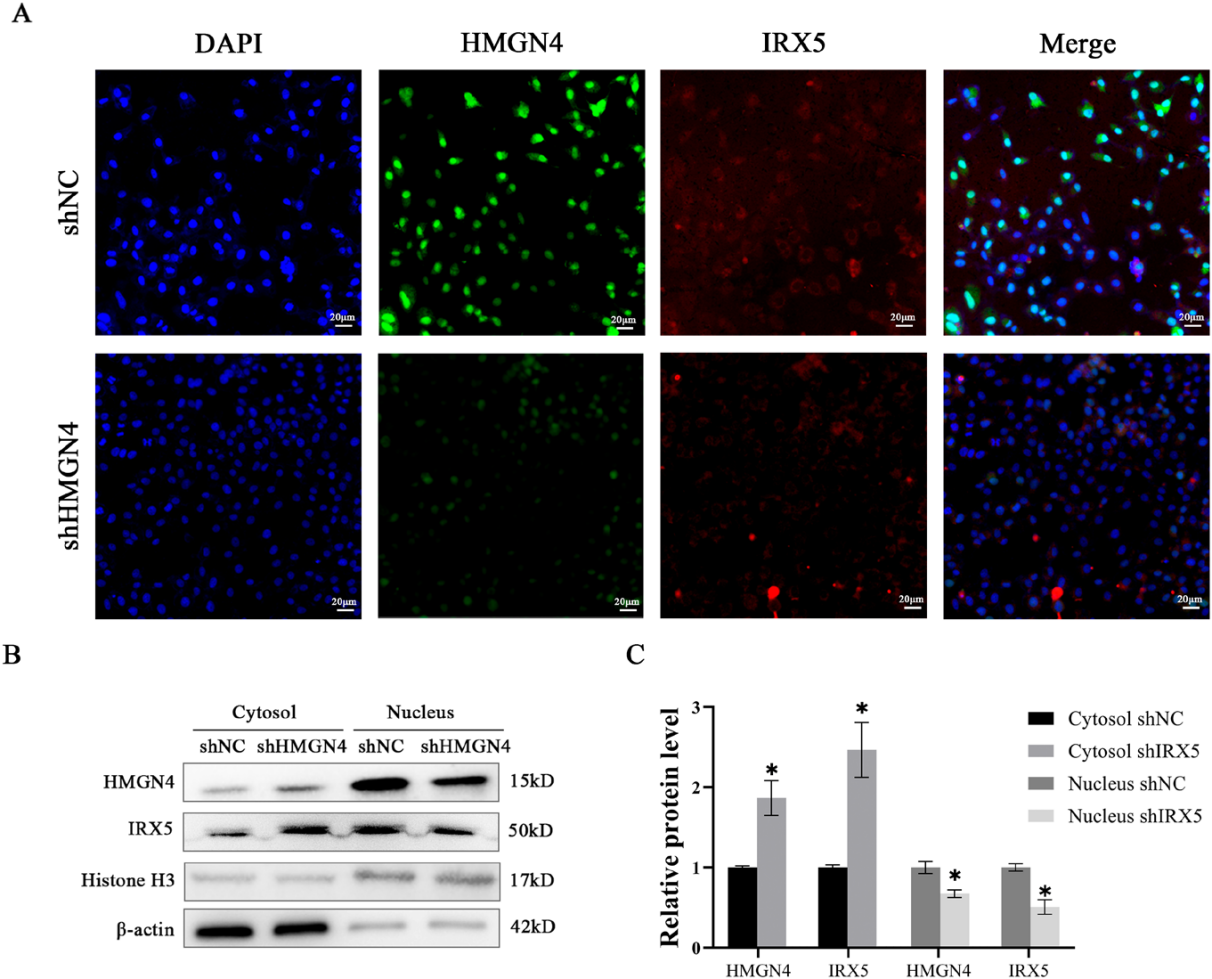
The amount of IRX5 in the nucleus decreased while the amount of IRX5 in the cytosol increased after HMGN4 knockdown. (A) Immunofluorescence staining of IRX5 (× immunofluorescence) and DAPI (× immunofluorescence) in the normal control and HMGN4‐knockdown groups. (B) Representative Western blot after cytosolic and nuclear fraction isolation. (C) Western blotting analysis. *indicates *p* < 0.05.

IRX5 is a transcription factor that usually regulates gene expression in the nucleus, but scholars have found that the transcription factor IRX5 lacks a nuclear localisation signal, which is contrary to the function of its transcription factor. HMGN4 is a chromatin‐related small nonhistone protein that contains a nuclear localisation signal and can shuttle between the cytoplasm and nucleus [19]. Therefore, we speculate that the two proteins have a synergistic regulatory relationship in the nucleus. To prove the relationship between these two proteins, immunofluorescence staining was performed and revealed that the level of IRX5 decreased after downregulation of HMGN4 expression (Figure 7A). Second, the levels of HMGN4 and IRX5 inside and outside the nucleus were measured, and the results showed that the level of IRX5 in the cytoplasm of HepG2‐shHMGN4 cells was increased, while the level of IRX5 in the nucleus was decreased (Figure 7B,C). These data show that HMGN4 assists IRX5 in entering the nucleus.

## 4 Discussion

Lipid metabolism is closely related to the progression and development of HCC [20, 21, 22]. Lipids play important roles in membrane composition, cell signalling, and energy sources [23], and studies have proven that both fatty acids (FAs) and cholesterol improve the activities of tumour cells. Seo Jieun et al. found that FFA‐induced FABP5 upregulation drives HCC progression through the HIF‐1‐induced reprogramming of lipid metabolism [24], while Wang Xiaobo et al. found that tubulin beta class I genes induce the expression of CYP27A1, leading to increased cholesterol levels and HCC progression [25]. We have studied the molecular function of IRX5 for a long time. We found that high expression of IRX5 leads to adverse outcomes of HCC10 [26]. However, whether IRX5 plays a role in lipid metabolism in HCC has not yet been reported. In recent years, some studies have shown that IRX5 may be associated with fat storage and consumption. After the specific knockout of IRX5 in hypertrophic chondrocytes, Tan et al. found that the absence of IRX5 promoted the differentiation of hypertrophic chondrocytes into adipocytes and increased adipose tissue in bone marrow [27], suggesting that IRX5 can inhibit adipogenic differentiation. CLAUSSNITZERM et al. found that IRX5 can regulate preadipocyte differentiation, decrease mitochondrial oxidation‐induced lipid accumulation, and reduce brown adipocyte numbers and transform them into energy‐storing white fat, resulting in an increase in fat storage [12]. YANG Q and other scholars found that interference with the expression of IRX5 in mouse adipocytes affects the energy balance of the body, resulting in weight loss and lipid storage in mice; however, increased IRX5 expression during preadipocyte differentiation notably disrupts the energy balance in mouse bodies, resulting in fat accumulation and obesity [13]. Bjune et al. found that IRX5‐knockout mice that consumed a high‐fat diet were protected from obesity [14]. To prove that IRX5 is involved in lipid metabolism, we performed substantial amounts of work. We proved that IRX5 plays a role in metabolism, and both fatty acid test results showed that IRX5 causes lipid accumulation in HCC and increases the concentrations of long‐chain fatty acids and triglycerides.

Several factors and enzymes play crucial roles in fatty acid synthesis, while SREBP1 is the core molecule that is involved in cellular lipid synthesis. SREBP1 activation directly triggers the transcription of the FASN gene, promotes the accumulation of lipid droplets, and promotes the proliferation of liver cancer cells [28]. Chen et al. found that SREBP1 is critical for TRIM21‐mediated lipogenesis inhibition in vitro and in vivo [29], whereas Zhang et al. found that the upregulation of pre‐mRNA processing factor 19 (PRP19) contributes to oesophageal squamous cell carcinoma progression by reprogramming SREBP1‐dependent fatty acid metabolism [30]. In this study, we found that IRX5 is highly related to SREBP1. After downregulating IRX5 in HepG2 and SMMC7721 cells, the expression level of SREBP1 was decreased. Thus, we hypothesise that downregulated IRX5 is upstream of SREBP1 and that IRX5 can positively regulate the expression of SREBP1 by functioning as a transcription factor.

Transcription factors (TFs) are a class of nuclear proteins that can regulate transcription by recognising cis‐acting elements in DNA sequences. Transcription factors enter the nucleus via a process that depends upon the recognition of their nuclear localisation signals (NLSs) and nuclear pore complexes, but only approximately 80% of the transcription factor sequences contain nuclear localisation signals. Although IRX5 is a transcription factor that is very important in physiological processes, as predicted by PSORT, cNLS and NucPred software, IRX5 lacks an NLS. In this study, after GST‐IRX5 pull‐down combined with HPLC/MS, we found that IRX5 might interact with HMGN4. HMGN4 is a member of the high mobility nucleosome binding protein family. The HMGNs family is a class of small nonhistone proteins that bind to nucleosomes. All the members of the HMGN family, which is a highly conserved protein family, have a two‐part nuclear localisation signal [31]. Studies have shown that HMGNs participate in many physiological processes, such as DNA repair [32], by specific binding to nucleosomes and preferential binding to chromatin regulatory sites [19]. A recent study showed that HMGN2, which is a member of the high mobility group nucleosome‐binding protein family, can promote the nuclear translocation of nuclear factor erythroid 2‐related factor 2 Nrf2 [33]. Thus, we assumed that by binding to HMGN4, IRX5 obtained a nuclear translocation signal and could be recognised and enter the nucleus, where it functions as a transcription factor. To prove this hypothesis, we performed many studies. We proved that IRX5 could bind to HMGN4 through immunoprecipitation and found that IRX5 and HMGN4 could be located in the nucleus at the same time through immunofluorescence. In addition, downregulating HMGN4 led to increased levels of IRX5 in the nucleus. We lack in vivo data, but we have ordered conditional liver‐specific IRX5‐knockout mice, which we believe will allow us to prove this hypothesis soon.

In general, we found that in patients with hepatocellular carcinoma, the expression of IRX5 is high, and the expression of IRX5 is positively correlated with the expression of SREBP1. IRX5 can enter the nucleus by interacting with HMGN4, upregulate the expression of genes that are related to fatty acid synthesis, and promote the proliferation and migration of hepatocellular carcinoma. These results suggest that IRX5‐HMGN4 may be an accurate prognostic indicator, and IRX5‐HMGN4 may also be a potential therapeutic target. Attention should be given to elucidating the mechanism underlying the upregulation of IRX5 and HMGN4 expression in liver cancer tissues, as well as other possible regulatory mechanisms, to provide a better theoretical basis for targeting IRX5‐HMGN4 molecules in the treatment of liver cancer.

## 5 Conclusion

In HCC, the expression of IRX5 in liver tissue is upregulated, which promotes the expression of proteins that are related to fatty acid biosynthesis and lipid droplet accumulation in hepatocytes. Furthermore, HMGN4 may facilitate the nuclear translocation of IRX5, thus promoting the expression of fatty acid biosynthesis‐related proteins.

## Author Contributions

**Liying Zhu:** validation (equal). **Yongjie Xu:** conceptualization (equal), data curation (equal). **Changyudong Huang:** validation (equal). **Chengcheng Li:** data curation (equal). **Yiqiong Zhang:** data curation (equal), methodology (equal). **Xing Li:** methodology (equal). **Wei Pan:** conceptualization (lead). **Zhu Zeng:** conceptualization (lead).

## Consent

We agree to publish the manuscript.

## Conflicts of Interest

The authors declare no conflicts of interest.

## Data Availability

This study encompasses all the data that was produced or examined, which are presented within this paper. Additional information can be obtained by reaching out to the lead author if needed. The patient‐related data underpinning the results of this research can be found within The Cancer Genome Atlas (TCGA) datasets, accessible at the following URL: https://portal.gdc.cancer.gov/repository.
